# A Generational Approach to Neurology Education: Engaging Generation Z Learners

**DOI:** 10.1212/NE9.0000000000200355

**Published:** 2026-08-17

**Authors:** Rafid Mustafa

**Affiliations:** From the Department of Neurology, Mayo Clinic College of Medicine and Science, Rochester, MN.

## Abstract

Neurophobia remains a persistent barrier in neurology education because learners often struggle to connect neuroanatomy, localization, examination findings, and clinical reasoning. At the same time, Generation Z learners now comprise much of undergraduate and graduate medical education and have trained in an environment shaped by ubiquitous mobile technology, short-form media, rapid information retrieval, and heightened attention to feedback, well-being, and belonging. This review synthesizes literature on neurophobia, Gen Z learner preferences, cognitive load, active learning, simulation, multimedia instruction, social media, artificial intelligence (AI), and recent *Neurology® Education* scholarship. It argues for a generationally informed, not generationally deterministic, approach: many recommended strategies predate Gen Z and benefit learners across age groups, but they are especially relevant to the current technologic and cultural learning environment. Practical strategies include selective flipped classrooms, microlearning with spaced retrieval, simulation and deliberate practice, coaching-oriented feedback, curated social media resources, supervised AI use, and psychologically safe teaching cultures. A critical, theory-grounded approach can help neurology educators preserve rigor while making the path into neurologic reasoning clearer.

## Introduction

Neurology is often experienced by learners as a high-difficulty discipline because it demands simultaneous integration of neuroanatomy, physiology, lesion localization, and probabilistic clinical reasoning. That challenge is not new. In 1994, Ralph Jozefowicz^1^ coined the term “neurophobia” to describe learners' fear of neurology when they cannot connect foundational science with bedside decision-making.

Subsequent reviews and meta-analyses have confirmed that neurophobia remains common among medical students and junior clinicians internationally. Recent contributors include insufficient integration of basic and clinical neuroscience, limited supervised examination practice, variable exposure to neurologic patients, and the perceived complexity of neurologic diagnosis.^2-4^

**Figure 1:**
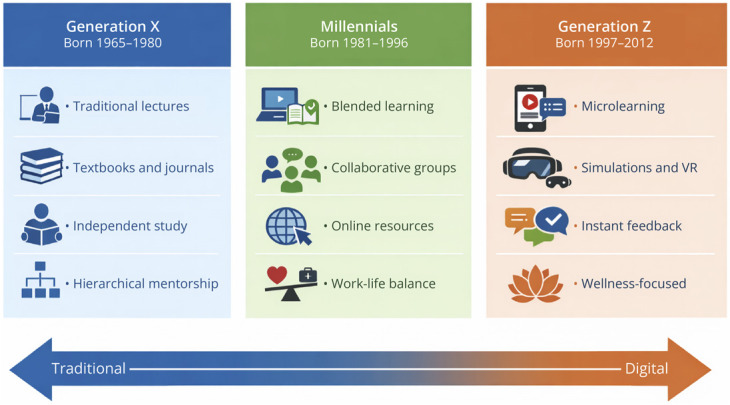
Generational Shifts in Medical Education and Implications for Neurology Training This figure summarizes broad shifts in the educational environment across Generation X (1965–1980), Millennials (1981–1996), and Generation Z (1997–2012). It should be read as a heuristic rather than a taxonomy of fixed learner traits. Generation X educators often trained in more information-scarce, textbook-centered and lecture-centered environments with hierarchical apprenticeship. Millennials reflect a transition toward blended learning, collaboration, and online resources. Generation Z learners have trained in a mobile, multimedia, social media, and emerging AI environment, with heightened expectations for feedback, relevance, and psychological safety. The gradient from traditional to digital highlights increasing interactivity and learner-centered delivery, but it does not imply that strategies such as feedback, scaffolding, simulation, or multimedia are uniquely beneficial to 1 generation. In neurology education, these shifts support combining cognitive load management, multimedia learning, situated practice, coaching, and technology governance to improve engagement and address neurophobia.

The urgency of addressing neurophobia now coincides with a shift in the dominant learner cohort in undergraduate medical education and early residency toward Generation Z (Gen Z), usually defined as individuals born in the late 1990s through the early 2010s. The educational importance of Gen Z is not that all learners in this cohort behave the same way; rather, they have trained during a period marked by mobile information access, social media, pandemic-related disruptions, and expanding attention to feedback, identity, and well-being (Figure 1).^5-8^

For neurology educators, the practical question is therefore not whether generational theory is perfect or whether Gen Z learners are categorically different from previous learners. It is how educators can use a cautious understanding of the current learning environment to make neurologic reasoning visible, reduce avoidable cognitive load, and preserve rigorous apprenticeship. This review argues that a generationally informed approach can help neurology educators reduce barriers to engagement when it is anchored in established educational theory rather than stereotypes.^2,3,5-10^

### Gen Z Learners in Medical Education

Scoping and narrative reviews in health professions education consistently describe Gen Z learners as digitally fluent, accustomed to rapid information retrieval, and comfortable navigating multimedia resources across devices. Many also tend to expect educational environments that are transparent, responsive, socially conscious, and aligned with real-world application.^5-7^ These patterns should be interpreted as contextual tendencies, not fixed traits. Digital fluency does not guarantee information appraisal, and frequent device use does not eliminate the need for structured guidance, bedside role modeling, or direct feedback.^8^

A useful comparison is with Generation X educators, many of whom trained in an information-scarce environment shaped by textbooks, scheduled lectures, and hierarchical apprenticeship. Gen Z learners train in an information-abundant environment shaped by search engines, video platforms, social media feeds, artificial intelligence (AI) tools, and asynchronous resources. The implication is not that Gen X and Gen Z have fundamentally different cognitive architectures; all learners benefit from clear goals, scaffolding, retrieval practice, feedback, and authentic clinical participation.^9-11^ The difference is that current learners' default study behaviors and expectations are often mediated by technologies that were peripheral or unavailable during earlier training.

Three design implications are especially relevant to neurology. First, concise and modular learning units can reduce the initial intimidation of neuroanatomy and localization when they are connected back to whole clinical problems. Second, active and experiential formats, such as case-based teaching, bedside examination, and simulation, help situate abstract knowledge in patient care. Third, frequent formative feedback and coaching are central to mastering the neurologic examination and diagnostic reasoning, regardless of generation.^5-7,9-11^

These observations should also be interpreted through the lens of learner diversity. Culture, socioeconomic background, language, disability, race, gender identity, immigration history, prior educational access, and institutional context all shape how learners experience technology, feedback, hierarchy, and psychological safety. Within-cohort variation may be greater than between-cohort variation. A generational lens is most useful when educators treat it as a prompt for local needs assessment rather than as a shortcut for knowing individual learners.^8^

### Why This Matters Particularly in Neurology

Neurology remains one of the specialties most likely to create a mismatch between learner effort and learner confidence. Compared with many other clerkship topics, novices may struggle to see the logic chain connecting symptoms, examination findings, neuroanatomy, localization, and management. When that chain is hidden, learners can interpret poor early performance as evidence that the subject itself is inaccessible.^1,2,4^

A Gen Z-informed strategy can make this chain more visible (Table 1), but the mechanism is educational rather than generational. Segmenting complex content into smaller units does not dilute rigor; it reduces extraneous load so learners can devote working memory to the intrinsic difficulty of localization and diagnosis. Similarly, well-designed combinations of words, diagrams, video, and clinical images can help learners connect symbolic representations, such as pathway diagrams, to concrete patient presentations.^9,10^

**Table 1:** Mapping Current Learner-Context Observations to Neurology Education Responses

Learner-context observation (not a universal trait)	Neurology teaching risk	Theory-grounded, high-yield response
Rapid digital access and information abundance	Learners may bypass foundational scaffolding or rely on fragmented, unvetted resources	Curated prework, mobile-accessible modules, guided resource lists, and source appraisal
Short-form media and attention ecology	Long didactics intensify cognitive overload in neuroanatomy and localization	Microlearning, segmentation, spaced retrieval, and reconnection to whole cases
Desire for relevance and authenticity	Abstract content may feel disconnected from patient care	Case-based sessions, situated practice, near-peer teaching, and bedside correlation
Feedback orientation and concern for psychological safety	Skill gaps in the neurologic examination may persist if feedback is delayed or shaming	Direct observation, coaching language, rapid formative feedback, and respectful challenge
Comfort with multimedia, social media, and AI	Learners may encounter inaccurate resources, privacy risks, or AI hallucinations	Annotated video, imaging-based discussion, social media literacy, supervised AI use, and verification norms
Substantial diversity within the cohort	Generational assumptions may alienate learners or obscure culture, access, disability, and identity	Local needs assessment, flexible pathways, accessible materials, and culturally responsive examples

Abbreviation: AI = artificial intelligence.

Simulation, bedside coaching, and case-based reasoning also reflect long-standing theory. They are compelling not simply because Gen Z learners prefer interactivity, but because situated learning places knowledge in the social and clinical context in which it will be used.^11^ Recent Neurology: Education reviews similarly position simulation as a way to provide contextualized, on-demand, competency-oriented neurology training, especially for neurologic emergencies, communication tasks, teamwork, and assessment.^12,13^

A practical example is a localization session that begins with a brief preclass diagram of the corticospinal tract, follows with a 2-minute video of an upper motor neuron pattern, and then asks learners to examine a standardized patient or video case while explaining the lesion aloud. The sequence uses a format that may feel familiar to digitally oriented learners, but its value rests on cognitive load management, multimedia signaling, retrieval, and situated clinical application.^9-11^

### Instructional Strategies for Engaging Gen Z Learners

Practical strategies for a Gen Z responsive curriculum are highlighted in Table 2.

**Table 2:** Practical Implementation Toolkit for a Gen Z-Responsive Neurology Curriculum

Educational goal	Example neurology application	Assessment signal	Resource intensity
Reduce cognitive overload	Preclass 8-min lesion-localization module plus in-class case discussion	Improved case explanation accuracy	Low
Increase examination confidence	Directly observed cranial nerve minisession with rapid feedback	Checklist completion and learner self-efficacy	Low
Improve emergency readiness	Stroke-code or status epilepticus simulation with debrief	Time-to-task and team communication behaviors	Moderate to high
Support longitudinal retention	Weekly mobile “neuro pearl” with retrieval quiz or brief case-based prompt	Quiz performance and retention over time	Low
Extend learning through open digital resources	Faculty-curated podcast, video, or case thread followed by source appraisal	Annotated takeaway and discussion of reliability, privacy, and bias	Low
Use AI safely for reasoning practice	Faculty-vetted chatbot patient or AI-generated differential diagnosis that learners must critique	Prompt log, source verification, and faculty-reviewed reasoning note	Low to moderate
Strengthen belonging and psychological safety	Resident-led orientation, mentorship pairing, feedback script, and explicit norms for uncertainty	Rotation evaluations and learner narrative feedback	Low to moderate
Scale implementation for smaller programs	Adapt predeveloped curricula from Neurology: Education or society-hosted repositories	Local adaptation plan and outcome audit	Low

Abbreviation: AI = artificial intelligence.

### Flipped Classroom Design

Flipped learning is attractive in neurology because it allows educators to move foundational exposition out of valuable face-to-face time and reserve in-person sessions for reasoning, interpretation, and application. In medical education broadly, systematic review evidence suggests that flipped classroom approaches are associated with favorable learner perceptions and, in some studies, improved knowledge outcomes, although the quality of the comparative evidence is mixed and heterogeneity is substantial.^14,15^

For neurology, the highest-yield use of flipping is selective rather than total. Short preclass modules can introduce vocabulary, pathway maps, or syndrome frameworks; classroom time can then focus on localizing cases, examining patients, or interpreting EEG, imaging, and video findings. A practical 60-minute session might assign an 8-minute preclass lesion-localization module, open with a 2-question retrieval quiz, and devote most of the session to small groups explaining why a hemiparesis pattern localizes to the cortex, internal capsule, brainstem, spinal cord, or peripheral nerve.^10,14,16^

To avoid simply shifting lecture time to homework, educators should keep prework brief, provide accountability through low-stakes retrieval, and explicitly reconnect preparatory content to bedside or case-based reasoning. This approach may feel efficient to Gen Z learners, but its benefit is best understood as a theory-driven method for preparing all learners to participate in higher-order clinical reasoning during synchronous time.^9-11,14^

### Microlearning and Spaced Reinforcement

Microlearning is especially promising in neurology because the field contains many threshold concepts that benefit from repeated exposure rather than 1-time saturation. Short lessons on cranial nerve patterns, brainstem syndromes, seizure semiology, visual field localization, or movement phenomenology can be sequenced longitudinally and reinforced through quizzes, flashcards, image-of-the-day style prompts, or brief video debriefs.^5-7^

The main educational advantage is not merely brevity. It is segmentation plus retrieval. Gen Z learners often seek concise content anyway; when educators intentionally harness that tendency using spaced retrieval and cumulative application, they can transform fragmented study habits into durable learning routines.^5-7^ Neurology-specific e-learning work, including rapid, high-yield platforms such as NeuroBytes, illustrates that short-form resources can be paired with assessment and continuing professional development rather than being limited to informal studying.^17^

### Simulation and Deliberate Practice

Simulation is a particularly strong fit for modern neurology education because it turns a cognitively intimidating subject into an observable sequence of actions. The broader health professions literature shows favorable effects of technology-enhanced simulation on knowledge, skills, behaviors, and patient-related outcomes. In neurology specifically, recent reviews describe simulation's utility for emergencies, procedures, teamwork, communication, and competency-based assessment.^12,13^

The value is not exclusive to Gen Z, but the format aligns with current learners' expectation for interactivity, explicit goals, and timely debriefing. Examples include a low-fidelity status epilepticus tabletop exercise, a stroke-code team simulation with time-to-task feedback, a standardized patient encounter for functional neurologic disorder counseling, or a brief bedside practice station for eliciting nuanced reflexes. It is important that simulation need not be high cost; role-play, structured cases, and low-fidelity task trainers can still provide meaningful deliberate practice when objectives and debriefing are well designed.^11-13^

### Multimedia and Visual Explanation

Neurology is inherently visual. Effective teaching often depends on showing rather than telling: eye movement videos, gait clips, seizure semiology, movement disorder phenomenology, lesion diagrams, vascular maps, EEG tracings, and serial imaging. Multimedia learning theory suggests that well-designed combinations of words and pictures can improve understanding when they reduce unnecessary processing, signal essential relationships, and avoid distracting decoration.^10^

For current learners, the implication is to replace some text-heavy exposition with intentionally designed visual explanations. A faculty member might pause a gait video to ask learners to predict the localization, annotate an MRI to link a lesion with examination findings, or compare normal and abnormal eye movements side by side before bedside practice. The goal is not flashy content but coherent signaling that improves transfer from preclinical content to clinical reasoning.^6,10^

### Social Media and Open Digital Scholarship

Social media can be incorporated as an instructional strategy when it is curated, time-limited, and tied to explicit learning objectives. Rather than asking learners to “go online,” educators can assign a faculty-vetted case thread, podcast episode, short video, or infographic and ask learners to identify the clinical question, appraise the source, and explain how the content changes their reasoning. Systematic reviews of social media in medical education suggest potential benefits for engagement, feedback, collaboration, and professional identity formation, but also emphasize variable study quality and the need for careful instructional design.^18,19^

In neurology, open digital scholarship can support longitudinal exposure to clinical signs that are hard to schedule during a short clerkship. For example, a resident can lead a 10-minute “social media journal club” in which learners compare an online tremor video, a peer-reviewed movement disorders resource, and a textbook description, then discuss patient privacy, consent, attribution, and misinformation. Podcasts and other asynchronous resources can also extend learning outside the clinical day, but they should be framed as supplements to, not substitutes for, supervised patient care and source verification.^20^

### AI and Chatbot-Supported Learning

Generative AI and large language models can be used as formal learning tools if educators define their role and limitations. Potential neurology applications include faculty-vetted chatbots that simulate a patient with headache, seizure, weakness, or diplopia; AI-generated practice questions keyed to a clerkship objective; and prompts that ask learners to compare 2 differential diagnoses or critique a localization explanation. These uses can create more opportunities for retrieval and rehearsal, especially when faculty time is limited.^21-23^

The same tools can also mislead. Artificial intelligence outputs may contain fabricated citations, biased assumptions, privacy risks, and plausible but incorrect reasoning. A safe neurology curriculum should therefore teach learners to use AI as a supervised practice partner rather than an authority: no protected health information, explicit disclosure of AI use when required, source verification against peer-reviewed or institutional materials, faculty review of generated cases, and reflection on where the model's reasoning diverges from clinical evidence. This framing turns AI literacy into part of professional formation rather than an unregulated shortcut.^21-23^

### Feedback, Coaching, and Near-Peer Teaching

Neurologic examination skill develops through repetition plus feedback. Current learners often expect fast, actionable input on performance, and neurology education can meet that need productively by normalizing brief coaching encounters: 1 observed examination maneuver, 1 diagnostic hypothesis, 1 focused piece of feedback, and 1 concrete next step.^5,6,24^

Resident-as-teacher models are especially useful here. Recent Neurology: Education scholarship highlights the educational importance of residents and reviews practical frameworks for feedback and teaching. Near-peer teachers can translate faculty expectations into accessible language while modeling how competent neurologists actually reason through uncertainty.^24^

Concrete resident-level practices include a 5-minute “localization huddle” before rounds, a bedside demonstration of 1 examination maneuver followed by learner return-demonstration, a 1-minute preceptor conversation after a consult, and a postcall debrief that names 1 reasoning strength and 1 next diagnostic step. A psychologically safe feedback script might be “what were you trying to assess? Here is what I observed. Here is what I would keep doing. Here is one adjustment for the next patient.” This keeps feedback specific while avoiding global judgments about ability or belonging.

### Well-Being, Belonging, and Psychological Safety

Any review of Gen Z learners that ignores well-being is incomplete. The literature repeatedly notes this cohort's interest in mentorship, psychological safety, work-life integration, identity, and meaning. Neurology training, meanwhile, exposes learners to high-stakes diagnoses, disability, death, diagnostic uncertainty, and emotionally intense family conversations. A climate that is dismissive, opaque, or humiliating will predictably worsen avoidance of the field.^5-8^

Engaging current learners therefore requires more than digital tools. It requires educational cultures that invite questions, treat uncertainty as a normal part of neurologic reasoning, and separate challenge from shame. Educators can protect psychological safety while maintaining rigor by setting expectations before observation, asking learners to self-assess first, giving feedback on behaviors rather than identity, and closing the loop with a specific plan for deliberate practice.^5-8,24^

### Adapting Strategies Across the UME-to-GME Continuum

The same strategy should look different for medical students, residents, and fellows. Medical students often need vocabulary, anatomy scaffolding, low-stakes examination practice, and reassurance that early localization difficulty is normal. For them, flipped modules, short videos, and structured bedside coaching should emphasize recognition of common patterns and confidence with core examination maneuvers.

Residents and fellows require progressive autonomy, efficiency, and specialty-specific reasoning. For residents, simulation can focus on emergency leadership, consult triage, and teaching medical students; AI-supported cases can be used to practice differential diagnosis while explicitly auditing model errors. Fellows may benefit from advanced multimedia libraries, peer teaching, and leadership roles in curriculum design. Differentiating implementation by training level prevents a “1-size-fits-all Gen Z curriculum” and keeps the focus on developmental needs.

### Pitfalls and Limitations of a Generational Approach

Generational framing can become simplistic if it is treated as destiny rather than context. It may create generational othering, in which educators describe learners as fundamentally different, deficient, or fragile, or generational essentialism, in which individuals are presumed to share preferences because of birth year. Not every learner values technology to the same degree, and some observed “Gen Z preferences” may instead reflect broader shifts in higher education, internet use, social media, pandemic-era training, and the availability of digital tools. Educators should therefore avoid using generational language to excuse poor self-direction or to stereotype trainees as needing entertainment rather than rigor.^8^

A second pitfall is technologic solutionism. Digital tools are helpful only when they solve a real instructional problem. A weak localization curriculum does not become strong because it is delivered by app, video, social media post, or AI chatbot. Similarly, excessive fragmentation into tiny learning objects can undermine coherence if educators fail to continually reconnect modules to the full neurologic problem-solving process.^6,10,14^

Finally, the evidence base is uneven. The literature on Gen Z in medicine is still developing, and many claims remain descriptive rather than causal. It is safer to view generational insights as a pragmatic layer placed over stronger educational principles—cognitive load management, multimedia design, situated learning, active learning, deliberate practice, feedback, and psychological safety—than as a standalone theory of instruction.^5-7,12,14^

Equity should also be explicit. Technology access, disability accommodations, bandwidth, language, cultural norms around feedback, and prior exposure to neurology vary widely. Culturally responsive implementation may require offline alternatives, captioned media, flexible participation formats, transparent expectations, and examples that reflect diverse patient populations. A local learner needs assessment is more defensible than assuming that all Gen Z learners want the same tools.

## Future Directions

The next phase of neurology education scholarship should test not simply whether learners “like” certain strategies, but whether these strategies improve durable learning, clinical performance, professional identity formation, and patient care. Recent Neurology: Education work on simulation, resident teaching, EEG education, peer review, and curriculum innovation signals a maturing field that is increasingly prepared to ask more rigorous implementation and outcomes questions.^13,16,24-26^

Four research priorities stand out. First, studies should compare theory-driven combinations of modalities—for example, microlearning plus simulation vs lecture plus case discussion—rather than evaluating isolated tools. Second, outcome measures should extend beyond satisfaction toward performance, retention, transfer, and learner belonging. Third, interventions should be designed with feasibility in mind so that smaller programs can adapt them without requiring major financial investment. Shared curricular repositories, including Neurology: Education's curriculum resource repository and society-hosted collections, may help reduce duplication and resource costs. Fourth, new technologies should be studied through educational theory and implementation science. Social media and AI tools require governance around privacy, attribution, misinformation, bias, accessibility, and faculty oversight. Research should examine not only whether these tools increase engagement but also how they affect reasoning quality, equity, professionalism, and the transition from Unergraduate Medical Education to Graduate Medical Education.^12,13,16,25^

## Conclusions

A generational approach to neurology education is most useful when it is concrete, critical, and evidence informed. Gen Z learners are not a monolith, and the strategies emphasized here are not uniquely beneficial to 1 birth cohort. Their value lies in the alignment between contemporary learners' technologic and cultural context and enduring educational principles: scaffolding, cognitive load management, retrieval, situated practice, feedback, and psychological safety. In a specialty still burdened by neurophobia, that alignment offers an opportunity not to make neurology easier, but to make the path into neurologic reasoning clearer. When educators combine curricular segmentation, case-based application, simulation, curated digital resources, supervised AI use, coaching, and belonging, they can preserve the discipline's rigor while improving learner engagement and confidence.

## Glossary

AI: artificial intelligence
Gen Z: Generation Z
